# Antibiotic prescribing practices and antibiotic use quality indicators in Luang Prabang, Lao PDR: a point prevalence survey in a tertiary care hospital

**DOI:** 10.1186/s12879-024-09614-4

**Published:** 2024-08-13

**Authors:** Christelle Elias, Nay Thi Ha, Onanong Sengvilaipaserth, Athip Phaychith, Vilada Chansamouth, Valy Phongsavath, Bounxou Keohavong, Khamsay Detleuxay, Phaylinh Maniphonh, Thongphout Soukhaseum, Philippe Vanhems, François-Xavier Babin

**Affiliations:** 1 Service d’Hygiène, Epidémiologie, Infectiovigilance et Prévention, Hospices Civils de Lyon, Lyon, France; 2 Équipe Santé Publique, Epidémiologie et Eco-évolution des Maladies Infectieuses (PHE 3 ID), Centre International de Recherche en Infectiologie (CIRI), Université de Lyon, Inserm, U1111, Université Claude Bernard Lyon 1, CNRS, UMR5308, ENS de Lyon, Lyon, France; 3 Fondation Mérieux, South-East Asia Regional Office, Vientiane, Laos; 4 Microbiology Laboratory, Mahosot Hospital, Vientiane, Laos; 5 Lao-Oxford-Mahosot Hospital-Wellcome Trust Research Unit (LOMWRU), Mahosot Hospital, Vientiane, Laos; 6 Centre for Tropical Medicine and Global Health, Nuffield Department of Medicine, University of Oxford, Oxford, UK; 7 Luang Prabang Hospital, Luang Prabang, Laos; 8 Food and Drug Administration, Vientiane, Laos; 9 Department of Healthcare and Rehabilitation, Ministry of Health, Vientiane, Laos; 10 Fondation Mérieux, Lyon, France; 11 Groupement Hospitalier Sud, Unité d’Hygiène, Epidémiologie, Prévention - Bâtiment 1 165 Chemin du Grand Revoyet , Pierre Bénite Cedex, 69 495 France

**Keywords:** Antibiotic, Antibiotic stewardship, Guidelines, LMICs, Antibiotic resistance

## Abstract

**Context:**

The increase and global dissemination of antibiotic resistance limit the use of antibiotics to prevent and treat infections. Implementing antibiotic stewardship programs guided by local data on prescription profiles is a useful strategy to reduce the burden of antibiotic resistance. The aim was to determine the prevalence of antibiotic use and guideline compliance at Luang Prabang provincial hospital, Lao PDR.

**Methods:**

A point prevalence survey of antibiotics was conducted among hospitalized patients admitted to Luang Prabang hospital (204 beds) in Lao PDR on May 25, 2023. All patients presenting at 8:00 AM were eligible. Sociodemographic data, indications for antibiotic use, and antibiotic prescriptions were collected from medical records using a paper-based questionnaire and entered into an electronic platform following WHO methodology. The prevalence of antibiotic use was determined.

**Results:**

Out of the 102 patients included, 60(58.8%) were undergoing antibiotic treatment, of which 33(55.0%) received combination therapy, and 7(10.5%) had two indications for antibiotic use. The highest prevalence was in the surgical ward (14/15, 93%) followed by general paediatrics (18/27, 67%). Out of the 100 antibiotic prescriptions, 47(47%) were for community-acquired infections, 26(26%) for surgical prophylaxis, 13(13%) for hospital-acquired infections and 5(5%) for medical prophylaxis. Twenty(20%) antibiotics were prescribed for obstetrics and gynaecology prophylaxis, 17(17%) for intra-abdominal infections, and 10(10.0%) for pneumonia treatment as well as bone, and joint infections. The main antibiotics prescribed were ceftriaxone 36(34.6%), metronidazole 18(17.3%), ampicillin 8(7.7%), and gentamicin 8(7.7%). Only 2(3%) samples were sent to the laboratory, one of which showed a positive culture for *Escherichia coli* Extended Spectrum β-Lactamase. According to the WHO Access Watch and Reserve classification, 55(52.9%) molecules belonged to the Access category, 47(49.1%) to the Watch category, and none to the Reserve category. Only 14.9% of antibiotic prescriptions were fully compliant with current guidelines.

**Conclusion:**

This study indicated a significant prevalence of antibiotic use and a very low compliance with guidelines at Luang Prabang provincial hospital, Lao PDR. This highlights an urgent need for comprehensive strategies at all levels to optimize antibiotic use in hospitals, emphasizing diagnostic improvements, and continued research to address the factors driving this excessive antibiotic usage and improve adherence to guidelines.

**Supplementary Information:**

The online version contains supplementary material available at 10.1186/s12879-024-09614-4.

## Introduction

Antimicrobial resistance (AMR) stands as one of the most pressing global health concerns of our time. This phenomenon occurs when bacteria, parasites, viruses, or fungi are exposed to antimicrobial substances but remain resilient, rendering these medicines ineffective. It is a natural adaptive process for organisms, allowing them to change and evolve. However, the alarming reality is that existing resistance patterns are not only persisting but also proliferating, while new patterns continue to emerge worldwide [1]. The menace of multidrug-resistant infections intensifies the challenges. Patients affected by these resistant bacteria face poorer clinical outcomes and an elevated risk of death [1]. Moreover, they consume a disproportionate share of healthcare resources compared to patients battling non-resistant strains of the same bacteria. Beyond the immediate health implications, AMR carries significant economic burdens [2]. In cases where infections resist first-line drugs, costlier therapies become necessary, often involving extended hospital stays and prolonged treatments. This not only escalates healthcare expenses but also contributes substantially to the economic strain caused by diseases. Understanding the complexity of AMR reveals a web of interconnected causes. Over-prescribing and over-dispensing of antimicrobial drugs by healthcare professionals, non-compliance with treatment regimens, the prevalence of low-quality medicines, incorrect prescriptions, and poor infection prevention and control practices in healthcare facilities are all contributing factors. AMR is not a challenge with a single root cause; rather, it thrives due to a combination of inappropriate antimicrobial use, lack of effective surveillance systems, insufficient infection prevention and control measures, and a paucity of accessible, affordable, and rapid diagnostic tests. In response to this growing crisis, the World Health Organization (WHO) took a pivotal step by formulating the Global Action Plan (GAP) in 2015 [3]. The primary objective of the GAP is to ensure the prolonged efficacy of infectious disease treatment and prevention through the responsible use of quality-assured medicines. A critical facet of combating AMR lies in optimizing the use of antimicrobial agents, particularly antibiotics. Antibiotics encompass a wide array of subclasses and substances, each with its spectrum of activity. They often target a range of pathogens, and bacteria can develop diverse resistance mechanisms against them. In essence, the inappropriate use of antibiotics is widespread, especially in low and middle-income countries (LMICs) where data on antibiotic consumption and use are scant. To address this information gap and inform effective policies, the WHO advocates for harmonized data collection and robust monitoring systems. Hospitals, with their diverse patient populations and high antibiotic usage, serve as invaluable settings for understanding antibiotic prescribing patterns. The concentration of patients needing antibiotics not only provides insights into prescription trends but also facilitates the implementation of targeted interventions to optimize antibiotic use. However, continuous data collection is a resource-intensive task, leading to the exploration of complementary surveillance methods such as point prevalence surveys (PPS) [4]. PPS, successfully employed in hospitals across the world, offer a snapshot of antibiotic use at a specific point in time [4]. The European Union and the United States have conducted regional surveys using this methodology. The WHO has endeavoured to develop a similar methodology tailored to the resources available in low and middle-income countries while ensuring comparability with data from high-income countries (HICs) [4]. This approach encourages standardization, allowing for meaningful comparisons of antibiotic use across various parameters, including time, hospitals, districts, countries, and regions. Research conducted in Western Pacific countries by the WHO, including Malaysia, Japan, China, and Vietnam, has revealed varying levels of antimicrobial usage in hospitals, ranging from 28.5 to 67.4% [5–8]. Encouraging appropriate antibiotic use across human, animal, and environmental sectors through coordinated activities within a One-health approach includes the initiation of antibiotic stewardship programs (ASPs) in healthcare settings [9, 10], with ASPs described as a coherent set of activities promoting the responsible use of antimicrobials [11]. Evaluating the quality of antibiotic prescribing patterns using PPS is a starting point for further improvements within ASPs.

The implementation of these PPS aligns seamlessly with national strategies, exemplified by the Lao People’s Democratic Republic (PDR) National Action Plan for AMR 2019–2023 and fits into the strategic objective 4, Action 4.2 [12]. By conducting these surveys, countries not only gather essential baseline data but also lay the foundation for tailored interventions and therefore improve local antibiotic prescription practices. These surveys can be periodically repeated, enabling countries to track progress, assess the impact of interventions, and ensure the effectiveness of their strategies. Indeed, Chansamouth et al. conducted repeated surveys over a 4-year period in Lao PDR, indicating a high prevalence of antibiotic use exceeding in majority 65% and proportions of compliance with guidelines below 30% [13].

In comparison with other healthcare facilities in Lao PDR [13], no estimation of the use of antibiotics has been done in Luang Prabang provincial hospitals. This study extends upon the previous survey rounds by performing a PPS at Luang Prabang provincial hospital, a tertiary care hospital located in the northern region of Lao PDR, as a component of a broader ASP, with the aim of establishing foundational data on all core variables outlined in the WHO PPS methodology [4]. The goal was to inform potential quality improvement initiatives that could be implemented in Luang Prabang and other hospitals across Lao PDR to improve antibiotic prescription practices. This dataset can be used as a foundation for developing and refining facility-specific antibiotic policies and provide basis for progress.

## Methods

### Study design and setting

A cross-sectional study using the WHO PPS protocol [4] on antibiotic use was conducted on May 25, 2023 in the provincial hospital of Luang Prabang, Lao PDR, a 204-bed tertiary teaching hospital. This hospital is an acute care hospital located in the suburban area of the city of Luang Prabang (population 70,000 inhabitants), forth city in Lao PDR in the Luang Prabang province (467 000 population in 2020). The provincial hospital of Luang Prabang encompasses an adult hospital (150 beds), as well as a 54-bed paediatric hospital (age limit 15 years old) and is equipped with one Intensive Care Unit, one infectious diseases ward, one surgical ward, one gynaecology and obstetrics ward, one internal medicine, one paediatrics and one neonatal unit with a pharmacy and a laboratory. In 2023, 10 232 patients were admitted amounting to 40 011 patient bed days. As described in the WHO protocol, we only included inpatient wards in this survey and we excluded emergency departments and day surgery wards. Data were collected for inpatients admitted to all hospital wards and present at 8.00 a.m. on the day of the survey (denominator). Detailed patient and antibiotic information was collected for patients who received an antibiotic at 8:00 a.m. on the day of the PPS (numerator).

### Data collection

Six auditors (three local physicians, two epidemiologists, one microbiologist) conducted this one-day survey, during which all included wards had to be audited once. The auditors were trained on the methodology and data collection prior to the start of the survey. Three forms were completed where the first one gathered hospital-level data, the second collected ward-level data such as the total number of inpatients and the last collected patient-level data. Data for the hospital-level characteristics and hospital questionnaire were obtained from the hospital management department. Ward-level data were obtained from each visited ward. For each patient who was prescribed and received at least one antibiotic at 8:00 a.m. on the day of the PPS, we gathered data including baseline patient characteristics (age, gender, date of admission, surgery since admission and presence of invasive devices), the prescribed antibiotics, their diagnosis according to a predefined list, and whether it concerned treatment for a community-acquired infection (CAI) or healthcare-associated infection (HAI) or prophylactic prescribing (for both medical or surgical prophylaxis). Medical prophylaxis included prevention of opportunistic infections in immunocompromised patients (e.g. HIV/AIDS patients), prevention of bacterial infections in patients with late-stage cirrhosis, upper gastrointestinal bleeding, and acute necrotizing pancreatitis. The nature and duration of antibiotic prophylaxis and curative antibiotic therapy were also collected. A microbiology form was also completed in case a sample was sent to the laboratory for bacterial confirmation and Antimicrobial Susceptibility Testing (AST) results. The WHO Anatomical Therapeutic Chemical (ATC) classification system (2022 version) [14] was used to classify the antibiotics as well as the Access, Watch and Reserve (AWaRe) classification developed by WHO [15, 16]. The survey team collected from the medical records. Data were recorded in a paper-based worksheet prior to entering them into an online-based application provided by WHO (https://amc-survey.voozanoo.net/amcsrv/) for anonymised data entry.

### Antibiotic use quality indicators

Quality indicators for antibiotic use encompass adherence to guidelines. In this study, Lao antimicrobial prescribing guidelines were used to assess guideline compliance in both adult and children [17, 18]. The definition of compliance was adapted from Willemsen et al. [19] and was evaluated based on: (i) the diagnosis of an infection, (ii) whether prophylaxis was indicated, (iii) whether an antibiotic was warranted based on the diagnosis (iv) the type of population (adult vs. paediatrics). Multiple antibiotics started on the same day for the same indication were considered combination therapy. Compliance to guidelines was categorized into three levels: full compliance, indicating the appropriate selection of antibiotics for an infection or prophylaxis; partial compliance, signifying at least one appropriate antibiotic choice in cases of combination therapy; and non-compliance, encompassing the administration of unnecessary antibiotics or deviations from national guidelines.

### Data analysis

This study served as a baseline exploratory analysis, aiming to generate hypotheses for future quality improvement initiatives on antibiotic use, given the limited number of patients included in the dataset. The prevalence rate of antibiotic use was defined as the proportion of total inpatients who were receiving antibiotics on the survey date divided by the total number of patient hospitalized at 8.00 a.m. We analysed the most commonly prescribed antibiotics and the reasons for use, categorized by ward type, indication for antibiotic use and diagnosis. Categorical variables were displayed as proportions. Descriptive and univariate statistical analyses were performed using Stata version 18.0 (StataCorp LLC, College Station, TX, USA). Univariate analyses were performed using the Chi-squared test or Fisher’s exact test for qualitative variables, and the Student’s t test or the Mann-Whitney test (depending on the nature of the variable or the size of the sample) for quantitative variables. Odds-ratio with 95% confidence intervals were estimated. No multivariate analysis was performed due to the exploratory nature of this survey and the small sample size. A significance level of less than 0.05 is considered associated.

## Results

**Fig. 1:**
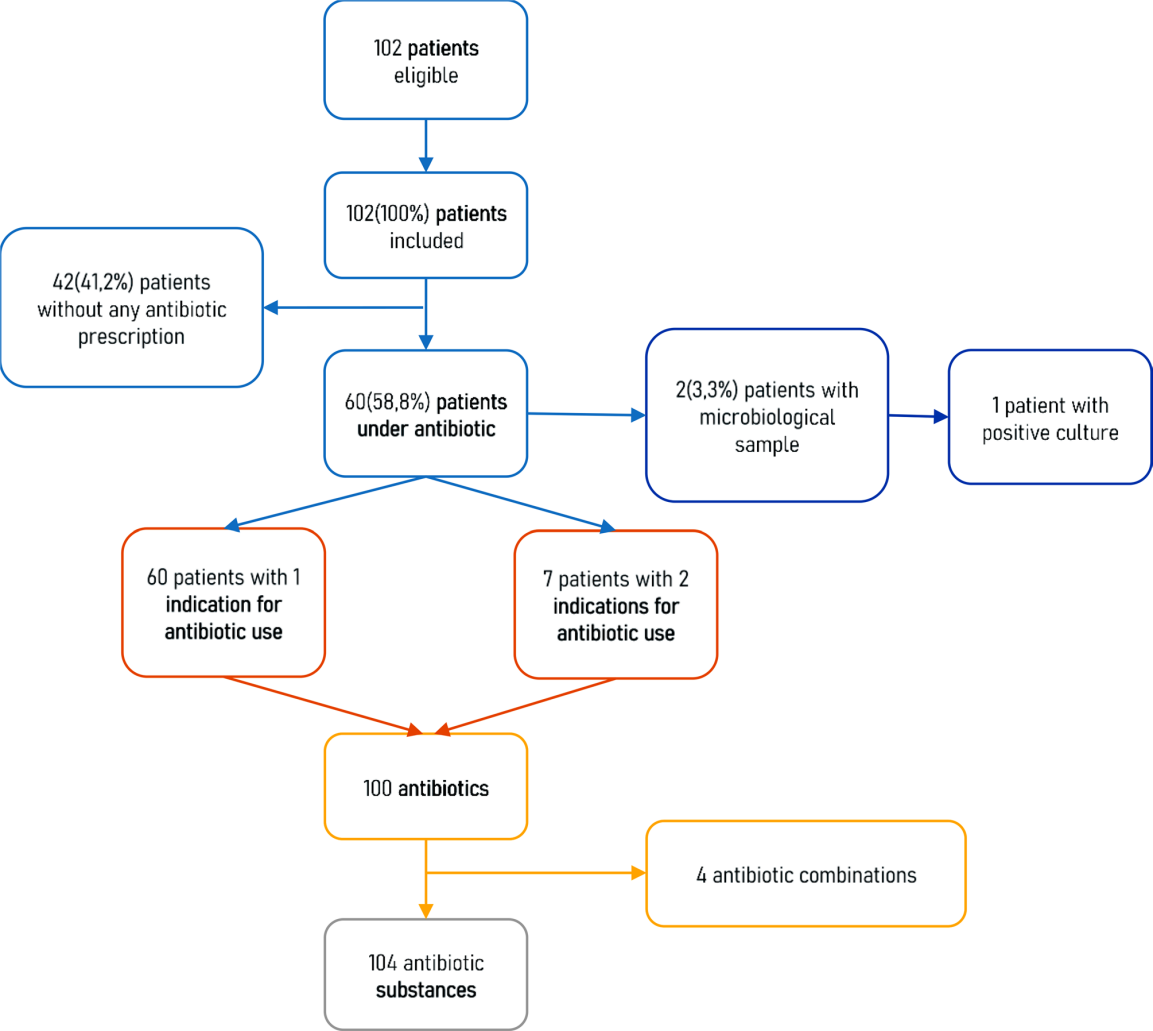
Flow chart of the data collected, Point Prevalence Survey on antibiotic use, Luang Prabang provincial hospital, Lao PDR, 2023

On May 25, 2023, a total of 102 patients were included in the survey with 58 (56.9%) at Luang Prabang adult hospital and 44 (43.1%) at Luang Prabang children’s hospital. Antibiotics were prescribed in 60 (58.8%) of the patients with 67 indications for antibiotic treatment. Twenty seven (45.0%) inpatients received one antibiotic, 25 (41.7%) received two antibiotics and 8 (13.3%) received three antibiotics. The highest prevalence of antibiotic use was in the general surgery ward (14/15, 93.3%) followed by the general pediatric ward (18/25, 66.7%). Only two (3%) samples were sent to the bacteriology laboratory, of which one showed a positive bacterial culture for *Escherichia coli* Extended Spectrum β-Lactamase (ESBL). Figure 1 shows the flow chart of the survey. Table 1 provides baseline characteristics of the data collected in the survey. The sex ratio was equal to 1.08 with a median age of 32.5 years old, Interquartile range (IQR)[27.5–52] in the adult population and 10 years old, IQR [0–15] in the pediatric population. The prevalence of antibiotic use did not differ by gender but differed significantly with patients who underwent surgery since hospital admission (*p* < 0.001) and by type of ward (*p* = 0.001).

### Antibiotic use and prescriptions patterns

Table 2 offers insight into the classification of antibiotics prescribed by ATC therapeutic subgroup and chemical subgroup. In total, 100 antibiotics were prescribed of these, third-generation cephalosporins accounted for 41 (41.0%) of all antibiotic prescriptions. The four main antibiotics prescribed at the hospital were respectively ceftriaxone (36, 36.0%), metronidazole (18, 18.0%), ampicillin (8, 8.0%), and gentamicin (8, 8.0%). The prevalence of vancomycin and meropenem use was 4 (4.0%) and 3 (3.0%), respectively. Eighty two (82%) antibiotic prescriptions were administered parenterally. Also, more than one fifth of the molecules were broad-spectrum antibiotics (81, 81.0%). Ceftriaxone was the most prevalent antibiotic prescribed in the gynecology ward, ICU, general medicine and surgery ward. Sulfamethoxazole-trimethoprim was the most prevalent antibiotic prescribed in the infectious diseases ward, as a prophylactic treatement against pneumocystosis and toxoplasmosis for HIV patients. Ampicillin was the most prevalent antibiotic prescribed in neonatalogy ward. According to the WHO AWaRe classification, 55 (55.0%) molecules belonged to the Access category, 47 (47.0%) to the Watch category, and none to the Reserve category. Out of the 100 prescriptions, 47 (47.0%) were for community-acquired infections, 26 (26.0%) for surgical prophylaxis, 13 (13.0%) for hospital-acquired infections involving mainly clinical sepsis, 5 (38.5%) and 5 (5.0%) for medical prophylaxis. Furthermore, among the total antibiotic prescriptions, 20 (20.0%) were for obstetrics and gynaecology prophylaxis, 17 (17.0%) were for intra-abdominal infections, of which 12 (70.6%) were community-acquired and 10 (10.0%) were for pneumonia treatment as well as bone, and joint infections (Table 3). More than half of the antibiotics (58, 58.0%) were prescribed for the adult population (Supplementary material 1).

Table 4 demonstrates the distribution of antibiotic by ATC Code and duration of antibiotic use in surgical prophylaxis. Cefazolin (ATC Code: J01DB04) was prescribed once, exclusively in septic arthritis. Ceftriaxone (ATC Code: J01DD04) together with Metronidazole (ATC Code: J01XD01) and Gentamicin (ATC Code: J01GB03) were the most commonly prescribed antibiotics in obstetrics and gynaecology surgical prophylaxis with multiple doses administered in more than one day.

### Antibiotic prescribing quality indicator

**Fig. 2:**
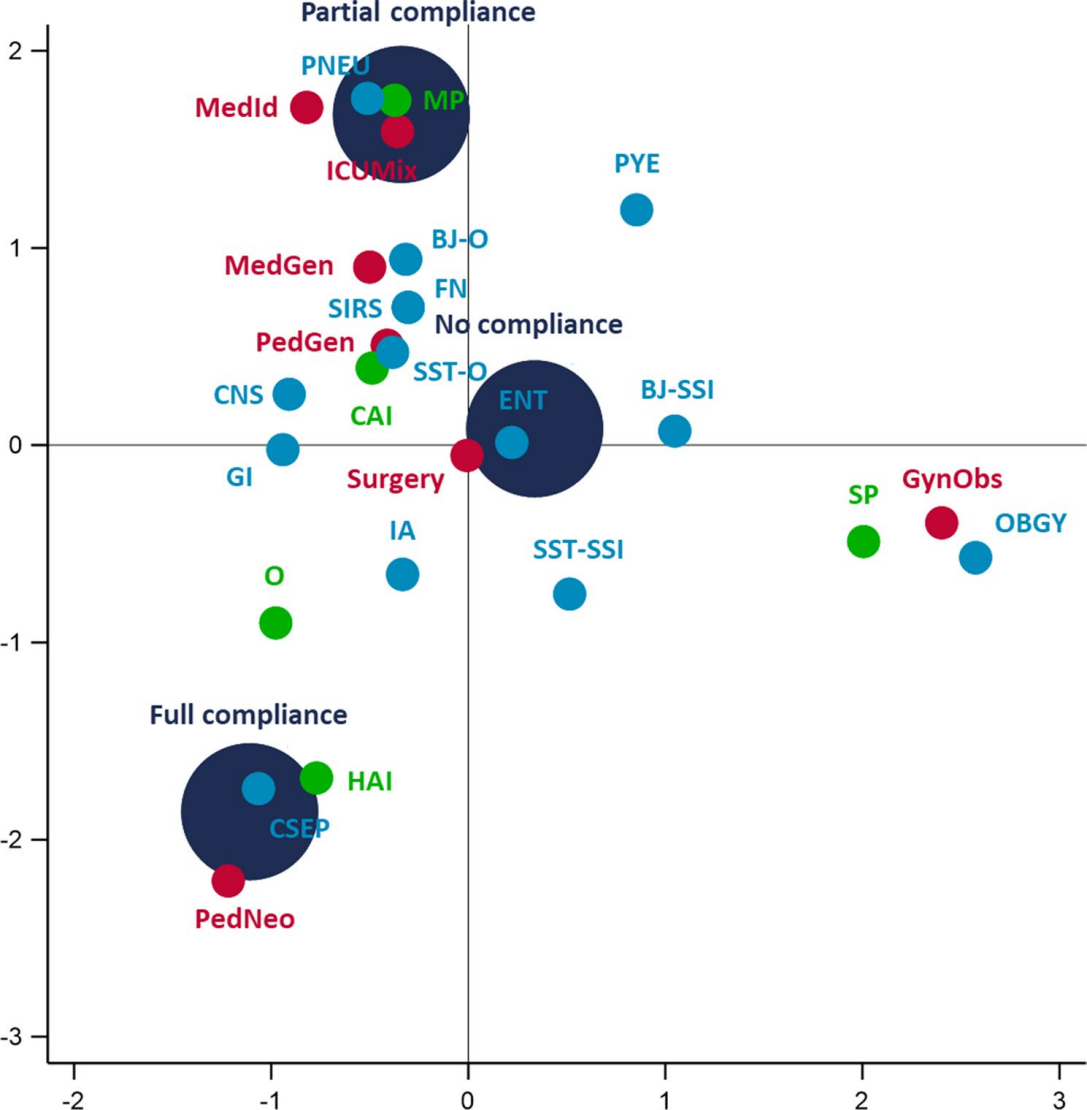
Multiple correspondence analysis coordinate plot of guidelines compliance by indication, diagnosis and type of ward. Obs: Obstretrics and Gynecology ward; ICUMix: Intensive Care Unit; MedGen: Internal Medicine ward; MedId: Infectious Disease ward; PedGen: Paediatrics ward; PedNeo: Neonatology ward; CAI: Community-acquired infection; HAI: Hospital-acquired infection; MP: Medical prophylaxis; SP: Surgical prophylaxis; OBGY: Obstetric or gynaecological infections; IA: Intra-abdominal sepsis, including hepatobiliary; CSEP: Clinical sepsis (suspected bloodstream infection without lab confirmation/results are not available, no blood cultures collected or negative blood culture), excluding febrile neutropenia; PNEU: Pneumonia; BJ-O: Septic arthritis, osteomyelitis, not related to surgery; SST-O: Cellulitis, wound, deep soft tissue not involving bone, not related to surgery; CNS: Infections of the central nervous system; SST-SSI: Surgical site infection involving skin or soft tissue but not bone; GI: Gastrointestinal infections (e.g. salmonellosis, antibiotic-associated diarrhoea); ENT: Infections of ear, nose, throat, larynx and mouth; FN: Febrile neutropenia or other form of manifestation of infection in immunocompromised host (e.g. HIV, chemotherapy, etc.) with no clear anatomical site; BJ-SSI : Septic arthritis, osteomyelitis of surgical site; PYE: Symptomatic upper urinary tract infection (e.g. pyelonephritis); SIRS: Systemic inflammatory response with no clear anatomical site

Table 5 presents compliance rates with antibiotic prescribing guidelines for different diagnoses in pediatric and adult patients. Sixty one (91.0%) antibiotic prescriptions were assessed and 6 (8.9%) were not due to missing or undefined diagnosis. Overall, 9 (14.8%) antibiotic prescriptions were fully compliant, 10 (16.4%) were partially compliant with guidelines and 42 (68.8%) antibiotic prescriptions were not compliant. Compliance (partial and full) with guidelines had a higher proportion in pediatric wards (36.6%) than in adults wards (25.8%). Antibiotic treatments for central nervous system infections and clinical sepsis were the most compliant antibiotic prescriptions. However, none of antibiotic prescription were compliant in obstetric infections, febrile neutropenia, wound surgical site infections, surgical site infection of bone and joint or Systemic Inflammatory Response Syndrome. The multiple correspondence analysis plot in Fig. 2 illustrated that antibiotic prescriptions for heathcare-associated clinical sepsis in neonatalogy were the most compliant with guidelines whereas surgical prophylaxis in the prevention of surgical site infection in the gynecology and obstetrics ward appeared to be no compliant with guidelines. A prescription of ceftriaxone (OR = 0.22, IC95%[0.07–0.73], *p* < 0.039) and the Watch group of the AWaRe classification (ref : Access group, OR = 0.23, IC95%[0.07–0.74], *p* = 0.049,) were significantly associated with a low compliance with guidelines, compared to an ampicillin prescription which was associated with a significantly higher compliance (OR = 9.23, IC95% [1.66–51.46], *p* = 0.011) with guidelines.

**Table 1:** Description of the data, Point Prevalence Survey on antibiotic use, Luang Prabang provincial hospital, Lao PDR, 2023

Variables	*n*	%
**Number of screened hospital charts**	102	(100)
**Number of patients with antibiotics**	60	(58.8)
**Type of ward**		
Pediatrics	27	(26.5)
Internal medicine	24	(23.5)
Obstetrics and Gynaecology	21	(20.6)
Surgery	15	(14.7)
Neonatalogy	11	(10.8)
Intensive Care Unit	2	(2)
Infectious diseases ward	2	(2)
**Gender**		
Men	53	(52.0)
Women	49	(48,0)
**Median age [IQR] in years**	28	[15–43]
In the adult population	33	[28–52]
In the pediatric population	10	[0–15]
**Age category**		
≤ 1 year	15	(14.7)
> 1–5 years	3	(2.9)
> 5–15 years	20	(19.6)
> 15 years	64	(62.7)
**Median delay between date of admission and date of survey [IQR] in days**	2	[1–5]
**History of surgery since hospital admission**	21	(20.6)
**Invasive devices**		
Peripheric vascular catheter	68	(66.7)
Urinary catheter	2	(2.0)
Intubation	1	(1.0)
Central vascular catheter	0	

IQR : Interquartile range

**Table 2:** Classification of antibiotics prescribed by Anatomical Therapeutic Chemical classification system therapeutic subgroup and chemical subgroup, Point Prevalence Survey on antibiotic use, Luang Prabang hospital, Lao PDR, 2023

Antibacterial for systemic use	Spectrum	ATC code	AWaRe	Total		GynObs		ICUMix		MedGen		MedId		PedGen		PedNeo		Surgery	
				*n*	%	*n*	%	*n*	%	*n*	%	*n*	%	*n*	%	*n*	%	*n*	%
Ceftriaxone	Broad	J01DD04	Watch	36	(36.0)	10	(43.5)	1	(100)	5	(71.4)	1	(25.0)	7	(25.0)	1	(7.1)	11	(47.8)
Metronidazole (IV)	Broad	J01XD01	Access	17	(17.0)	6	(26.1)	0		1	(14.3)	0		1	(3.6)	2	(14.3)	7	(30.4)
Ampicillin	Narrow	J01CA01	Access	8	(8.0)	0		0		0		0		2	(7.1)	6	(42.9)	0	
Gentamicin	Broad	J01GB03	Access	8	(8.0)	5	(21.7)	0		0		0		0		1	(7.1)	2	(8.7)
Cloxacillin	Narrow	J01CF02	Access	6	(6.0)	0		0		0		0		6	(21.4)	0		0	
Cefotaxime	Broad	J01DD01	Watch	5	(5.0)	0		0		0		0		1	(3.6)	4	(28.6)	0	
Sulfamethoxazole/ Trimethoprim	Broad	J01EE01	Access	4	(4.0)	0		0		0		2	(50.0)	2	(7.1)	0		0	
Vancomycin (IV)	Broad	J01XA01	Watch	4	(4.0)	0		0		0		0		4	(14.3)	0		0	
Meropenem	Broad	J01DH02	Watch	3	(3.0)	0		0		0		0		1	(3.6)	0		2	(8.7)
Doxycycline	Broad	J01AA02	Access	2	(2.0)	1	(4.3)	0		0		1	(25.0)	0		0		0	
Cefalexin	Narrow	J01DB01	Access	2	(2.0)	1	(4.3)	0		0		0		1	(3.6)	0		0	
Amoxicillin	Narrow	J01CA04	Access	1	(1.0)	0		0		0		0		0		0		1	(4.3)
Phenoxymethylpenicillin	Narrow	J01CE02	Access	1	(1.0)	0		0		0		0		1	(3.6)	0		0	
Cefazolin	Narrow	J01DB04	Access	1	(1.0)	0		0		0		0		1	(3.6)	0		0	
Azithromycin	Broad	J01FA10	Watch	1	(1.0)	0		0		1	(14.3)	0		0		0		0	
Metronidazole (O)	Broad	P01AB01	Access	1	(1.0)	0		0		0		0		1	(3.6)	0		0	
Total				100	(100)	23	(100)	1	(100)	7	(100)	4	(100)	28	(100)	14	(100)	23	(100)

ATC code: Anatomical Therapeutic Chemical classification code; GynObs: Obstretrics and Gynecology ward; ICUMix: Intensive Care Unit; MedGen: Internal Medicine ward; MedId: Infectious Disease ward; PedGen: Paediatrics ward; PedNeo: Neonatology ward; IV: Intravenous route; O: Oral route

**Table 3:** Most prevalent diagnoses for antibiotic prescription by hospital, Point Prevalence Survey on antibiotic use, Luang Prabang hospital, Lao PDR, 2023

Diagnosis	Antibiotics		Therapeutic use				Prophylactic use				Other indication for antibiotic use	
	Total		CAI		HAI		MP		SP			
	*n*	%	*n*	%	*n*	%	*n*	%	*n*	%	*n*	%
OBGY	20	(20.0)	0		0		0		20	(76.9)	0	
IA	17	(17.0)	12	(25.5)	3	(23.1)	0		2	(7.7)	0	
CSEP	13	(13.0)	7	(14.9)	5	(38.5)	0		0		1	(11.1)
PNEU	10	(10.0)	8	(17.0)	0		2	(40.0)	0		0	
BJ-O	10	(10.0)	9	(19.1)	0		1	(20.0)	0		0	
SST-O	6	(6.0)	2	(4.3)	2	(15.4)	2	(40.0)	0		0	
CNS	4	(4.0)	4	(8.5)	0		0		0		0	
SST-SSI	4	(4.0)	0		2	(15.4)	0		2	(7.7)	0	
UND	4	(4.0)	0		0		0		0		4	(44.4)
GI	3	(3.0)	1	(2.1)	0		0		0		2	(22.2)
ENT	2	(2.0)	0		1	(7.7)	0		1	(3.8)	0	
FN	2	(2.0)	2	(4.3)	0		0		0		0	
BJ-SSI	1	(1.0)	0		0		0		1	(3.8)	0	
PYE	1	(1.0)	1	(2.1)	0		0		0		0	
SIRS	1	(1.0)	1	(2.1)	0		0		0		0	
Missing	2	(2.0)	0		0		0		0		2	(22.2)
Total	100		47		13		5		26		9	

CAI: Community-acquired infection; HAI: Hospital-acquired infection; MP: Medical prophylaxis; SP: Surgical prophylaxis; OBGY: Obstetric or gynaecological infections; IA: Intra-abdominal sepsis, including hepatobiliary; CSEP: Clinical sepsis (suspected bloodstream infection without lab confirmation/results are not available, no blood cultures collected or negative blood culture), excluding febrile neutropenia; PNEU: Pneumonia; BJ-O: Septic arthritis, osteomyelitis, not related to surgery; SST-O: Cellulitis, wound, deep soft tissue not involving bone, not related to surgery; CNS: Infections of the central nervous system; SST-SSI: Surgical site infection involving skin or soft tissue but not bone; UND: Completely undefined; site with no systemic inflammation; GI: Gastrointestinal infections (e.g. salmonellosis, antibiotic-associated diarrhoea); ENT: Infections of ear, nose, throat, larynx and mouth; FN: Febrile neutropenia or other form of manifestation of infection in immunocompromised host (e.g. HIV, chemotherapy, etc.) with no clear anatomical site; BJ-SSI : Septic arthritis, osteomyelitis of surgical site; PYE: Symptomatic upper urinary tract infection (e.g. pyelonephritis); SIRS: Systemic inflammatory response with no clear anatomical site

**Table 4:** Distribution of antibiotic by ATC code and duration of antibiotic use in surgical prophylaxis, Point Prevalence Survey on antibiotic use, Luang Prabang hospital, Lao PDR, 2023

Antibacterial for systemic use	ATC Code	Total		BJ-SSI		ENT		IA		OBGY		SST-SSI	
				One dose		Multiple doses on more than one day		Multiple doses on more than one day		Multiple doses on more than one day		Multiple doses on more than one day	
		*n*	%	*n*	%	*n*	%	*n*	%	*n*	%	*n*	%
Cefazolin	J01DB04	1	(3.8)	1	(100)								
Ceftriaxone	J01DD04	12	(46.2)			1	(100)	1	(50.0)	9	(45.0)	1	(50.0)
Gentamicine	J01GB03	6	(23.1)							5	(25.0)	1	(50.0)
Metronidazole	J01XD01	7	(26.9)					1	(50.0)	6	(30.0)		
Total		26	(100)	1	(100)	1	(100)	2	(100)	20	(100)	2	(100)

ATC code: Anatomical Therapeutic Chemical classification code; BJ-SSI: Septic arthritis, osteomyelitis of surgical site; ENT: Infections of ear, nose, throat, larynx and mouth; IA: Intra-abdominal sepsis, including hepatobiliary; OBGY: Obstetric or gynaecological infections; SST-SSI: Surgical site infection involving skin or soft tissue but not bone

**Table 5:** Guidelines compliance assessment of antibiotic prescriptions (*n* = 61)

Diagnosis	Pediatrics						Adults						Total						
	None		Partial		Full		None		Partial		Full		None		Partial		Full		Total
	*n*	%	*n*	%	*n*	%	*n*	%	*n*	%	*n*	%	*n*	%	*n*	%	*n*	%	*n*
BJ-O	5	(83.3)	0		1	(16.7)	1	(100)	0		0		6	(85.7)	0		1	(14.3)	7
BJ-SSI	1	(100)	0		0		0		0		0		1	(100)	0		0		1
CNS	1	(50.0)	0		1	(50.0)	0		1	(100)	0		1	(33.3)	1	(33.3)	1	(33.3)	3
CSEP	4	(44.4)	5	(55.6)	0		0		0		0		4	(44.4)	5	(55.6)	0		9
ENT	1	(100)	0		0		0		0		1	(100)	1	(50.0)	0		1	(50.0)	2
FN	1	(100)	0		0		0		0		0		1	(100)	0		0		1
GI	0	0	1	(100)	0		1	(100)	0		0		1	(50.0)	1	(50.0)	0		2
IA	0	0	1	(100)	0		6	(75.0)	2	(25.0)	0		6	(66.7)	3	(33.3)	0		9
OBGY	0		0		0		9	(100)	0		0		9	(100)	0		0		9
PNEU	3	(75.0)	0		1	(25.0)	2	(40.0)	0		3	(60.0)	5	(55.6)	0		4	(44.4)	9
PYE	0		0		0		0		0		1	(100)	0		0		1	(100)	1
SIRS	1	(100)	0		0		0		0		0		1	(100)	0		0		1
SST-O	2	(66.7)	0		1	(33.3)	2	(100)	0		0		4	(80.0)	0		1	(20.0)	5
SST-SSI	0		0		0		2	(100)	0		0		2	(100)	0		0		2
Total	19	(63.3)	7	(23.3)	4	(13.3)	23	(74.2)	3	(9.7)	5	(16.1)	42	(68.9)	10	(16.4)	9	(14.8)	61

OBGY: Obstetric or gynaecological infections; IA: Intra-abdominal sepsis, including hepatobiliary; CSEP: Clinical sepsis (suspected bloodstream infection without lab confirmation/results are not available, no blood cultures collected or negative blood culture), excluding febrile neutropenia; PNEU: Pneumonia; BJ-O: Septic arthritis, osteomyelitis, not related to surgery; SST-O: Cellulitis, wound, deep soft tissue not involving bone, not related to surgery; CNS: Infections of the central nervous system; SST-SSI: Surgical site infection involving skin or soft tissue but not bone; UND: Completely undefined; site with no systemic inflammation; GI: Gastrointestinal infections (e.g. salmonellosis, antibiotic-associated diarrhoea); ENT: Infections of ear, nose, throat, larynx and mouth; FN: Febrile neutropenia or other form of manifestation of infection in immunocompromised host (e.g. HIV, chemotherapy, etc.) with no clear anatomical site; BJ-SSI : Septic arthritis, osteomyelitis of surgical site; PYE: Symptomatic upper urinary tract infection (e.g. pyelonephritis); SIRS: Systemic inflammatory response with no clear anatomical site

## Discussion

This study, the first using the WHO PPS methodology in Lao PDR indicated a prevalence of antibiotic use at Luang Prabang provincial hospital of 58.8%, which demonstrated lower results to surveys conducted in other hospitals in Lao PDR where the prevalence was equal to 69.7%, 74.3% and 72.9% in 2020, 2021 and 2022, respectively [20]. However, it’s worth noting that the overall prevalence of antibiotic use estimated at Luang Prabang provincial hospital exceeded the one reported in hospitals in high income countries where the prevalence does not reach 50% [21]. The proportion of antibiotic use varied from 37% in East and South Asian countries (29 hospitals) to 42% in nine west and central Asian countries (27 hospitals) [21]. The higher prevalence of antibiotic prescribing observed in this survey, as well as in surveys conducted in other hospitals in LMICs, in comparison to HICs, may be indicative of a greater burden of infectious diseases, more severe illnesses, or less stringent antibiotic prescribing policies.

Less than 15% of antibiotic prescriptions were fully compliant with current recommendations [17, 18], which was half as low as previous estimations in other healthcare facilities in Lao PDR [13], or to other Southeast Asian countries [6, 8]. Such low estimations might be explained by a lack of information about the guidelines, and by limited access to antibiotics in Luang Prabang hospital or by shortages which occurred at the time of the survey of ciprofloxacin, cephalexin and doxycycline which may have hampered the prescription of these drugs, recommended in the guidelines. There is clear evidence that adherence to guideline improves antibiotic use [22]. Better dissemination of the guidelines together with increased awareness, education and policy engagement might also assist prescribers in their clinical decision-making and therefore optimize the use of antibiotics.

Interestingly, this study revealed a higher proportion of antibiotic prescriptions for both medical and surgical prophylaxis compared to several other studies outside Lao PDR [23, 24]. However, this study did not delve into the reasons for antibiotic use for prophylactic purposes, warranting further investigation to determine whether the current proportion of antibiotic use allocated to prophylaxis is appropriate or necessitates intervention. Notably, prescriptions for treating hospital-acquired infections were lower in this study compared to other settings but associated with a better compliance with guidelines. Surprisingly, the majority of antibiotics at Luang Prabang hospitals were administered via the parenteral route. This pattern is consistent with previous estimations [13] and with a 2017 study conducted in Belgian acute care hospitals, where antibiotics were administered parenterally to 64.6% of patients [25]. It is likely that the high proportion of parenteral antibiotic administration at is partly attributed to the frequent use of ceftriaxone, metronidazole, and other parenteral antibiotics for which no equivalent oral formulations are available [26]. This prescribing pattern may also be attributed to the population’s belief in the superior efficacy of parenterally administered medicines. However, studies have shown that oral treatments can reduce the risk of hospital-acquired infections and offer both clinical and economic benefits [27]. Nonetheless, regular clinician review of the necessity for parenteral therapy could promote the increased use of oral treatment when appropriate and therefore reduce the burden of AMR.

The widespread use broad-spectrum antibiotics under the Watch category of the WHO AWaRe classification [16] – mainly third-generation cephalosporins –aligns with findings from other antibiotics use surveys conducted in Asian and African countries but contrasts with the higher use of narrow-spectrum penicillins and penicillin-enzyme inhibitor combinations observed in the 2015 Global-PPS [21]. High levels of use of third-generation cephalosporins meaningfully drive the increase in ESBL-producing Enterobacteriaceae, also in Lao PDR [28]. Chansamouth et al. showed that the proportion of ESBL-producing *Escherichia coli* among cultured isolates of *E.coli* in blood culture is increasing year on year, going from 7% in 2004 to 35% in 2016 [13]. One reason for the frequent prescription of broad-spectrum antibiotics in this study may be the high prevalence of antibiotic resistance among several key pathogens as well as the availability of these drugs in hospitals. The judicious use of broad-spectrum antibiotics may be supported by an increased utilization of high-quality clinical microbiology laboratory services [29]. These results can be employed collectively to inform empiric therapy guidelines within the hospital and to rationalize the treatment of individual patients [30]. Such guidelines ought to align with the recommendations provided by the WHO essential medicines list, which advocates for the use of antibiotics categorized under the Access group which typically exhibit a narrow spectrum of activity [16, 31].

The 2015 Global-PPS demonstrated that across 53 countries, 37.9% of therapeutic antimicrobial prescribing was based on microbiology results [21]. In contrast, in this survey, we found that less than 5%of prescriptions at Luang Prabang hospital were based on microbiology results highlighting how neglected laboratory services are in this setting. The high prevalence of broad-spectrum therapies may be linked to the limited use of diagnostic services at the hospitals. Therefore, we suggest strengthening clinical microbiology services, encouraging clinicians to utilize them, and preparing, disseminating, and regularly updating guidelines on antibiotic use to inform empiric treatment decisions at each hospital. Clinical practice guidelines have the potential to improve quality of care through improving decision making and antibiotic prescription. These guidelines are particularly important in areas with limited laboratory and specialist capacity [22].

International and national guidelines for surgical prophylaxis recommend the administration of a single dose of narrow-spectrum antibiotics within the 24-hour preoperative period [17, 32]. However, surgical prophylaxis at Luang Prabang hospital was often prescribed for durations exceeding one day and frequently involved the use of broad-spectrum antibiotics such as ceftriaxone and metronidazole. Prolonged surgical prophylaxis does not benefit the patient but increases the risk of antibiotic resistance and adverse events, including acute kidney injury and *Clostridioides difficile* infection [33]. We suggest that adopting and implementing surgical prophylaxis guidelines at Luang Prabang provincial hospital represents a vital opportunity to enhance patient safety and control AMR.

As many LMICs, Lao PDR struggles with implementing strategies to combat AMR, in particular in hospital settings. Antibiotic stewardship activities to promote the rational use of antimicrobials in hospitals remain scarce and to our knowledge no such activity was in place over this survey period in Luang Prabang provincial hospital. Strengthening and empowering hospital drug and therapeutics committees, along with pharmacists, to oversee antibiotic use and propose interventions for adherence to guidelines, would represent a significant step forward.

This study has several limitations. Firstly, due to the cross-sectional design, we collected only antibiotic prescribing data on each ward for a single day, making it difficult to ascertain whether the observed prescribing practices were representative of typical practices. Secondly, we did not measure the severity and acuity of infection, the duration of antibiotic use, or whether clinical staff altered the route of antimicrobial administration over time or adjusted antibiotic prescriptions based on microbiology test results after the ward surveys. Thirdly, guideline compliance was assessed in comparison with national guidelines, as hospital-specific treatment guidelines were not available. Also resistance patterns in Luang Prabang might differ from other healthcare facilities in Lao PDR. Adherence to guidelines for antibiotic use was determined solely by the selection of antibiotics, without considering dosage or duration, potentially leading to an overestimation of the appropriatness of antibiotic use. Finally, only mandatory variables of the WHO protocol were collected in this survey.

## Conclusion

This study revealed a prevalent use of antibiotics, particularly broad-spectrum ones with low adherence to guidelines. These findings underscore the importance of raising awareness and providing support for antibiotic stewardship program interventions in the hospital setting. The data collected in our survey can serve as a foundational reference point for a series of PPSs to track antibiotic prescribing patterns and gauge the impact of ASP interventions over time in Lao PDR. To ensure the effectiveness of ASPs, it is imperative to allocate resources for the review and development of evidence-based antibiotic prescribing guidelines adapted to local resistance patterns. Making standard guidelines available, endorsed by national health authorities, may instill confidence in clinicians regarding best practices, thereby enhancing the accuracy of diagnoses and antibiotic treatments. Additionally, there should be efforts to improve the documentation of antibiotic prescriptions, boost the utilization of microbiology testing, and provide support for microbiological laboratory services. Finally, the implementation and success of ASP interventions need to be thoroughly evaluated.

### Electronic supplementary material

Below is the link to the electronic supplementary material.

Supplementary Material 1

## Data Availability

The datasets used and/or analysed during the current study are available from the corresponding author upon reasonable request. The datasets were also shared with WHO Headquarters for global analysis.
