# Supplementary material for: Antibiotic prescribing practices and antibiotic use quality indicators in Luang Prabang, Lao PDR: a point prevalence survey in a tertiary care hospital
Source: BMC Infect Dis. 2024 Aug 13;24:818. doi: 10.1186/s12879-024-09614-4 (PMC11321149; doi:10.1186/s12879-024-09614-4)
Supplement: Supplementary file 1 — Supplementary Material 1 [file 12879_2024_9614_MOESM1_ESM.docx]

Table 3. Indication for antibiotic use by hospital, Point Prevalence Survey on antibiotic use, Luang Prabang hospital, Lao PDR, 2023

|  | **Antibiotics** | | **Therapeutic use** | | | | **Prophylactic use** | | | | **Other** | |
| --- | --- | --- | --- | --- | --- | --- | --- | --- | --- | --- | --- | --- |
|  | **Total** | | **CAI** | | **HAI** | | **MP** | | **SP** | |  | |
|  | **n** | **%** | **n** | **%** | **n** | **%** | **n** | **%** | **n** | **%** | **n** | **%** |
| **Adults** | 58 | (58) | 18 | (38.3) | 4 | (30.8) | 5 | (100) | 25 | (96.2) | 6 | (66.7) |
| **Children** | 42 | (42) | 29 | (61.7) | 9 | (69.2) | 0 |  | 1 | (3.8) | 3 | (33.3) |
| **Total** | 100 | (100) | 47 | (100) | 13 | (100) | 5 | (100) | 26 | (100) | 9 | (100) |

CAI: Community-acquired infection; HAI: Hospital-acquired infection; MP: Medical prophylaxis; SP: Surgical prophylaxis ; Other: indications that were not considered as therapeutic or prophylactic.
